# Data security crisis in universities: identification of key factors affecting data breach incidents

**DOI:** 10.1057/s41599-023-01757-0

**Published:** 2023-05-30

**Authors:** Jin Li, Wei Xiao, Chong Zhang

**Affiliations:** 1 School of Management, Xi’an Jiaotong University, Xi’an, China; 2 National Computer Network Emergency Response Technical Team/Coordination Center of China, Beijing, China

**Keywords:** Information systems and information technology, Education, Science, technology and society

## Abstract

The extremely complex and dynamic digital environments of universities make them highly vulnerable to the risk of data breaches. This study empirically investigated the factors influencing data breach risks in the context of higher education, according to *crime opportunity theory* and *routine activity theory*. The data consisted of university samples from China and were collected mainly from the Chinese Education Industry Vulnerability Reporting Platform. After applying Poisson regression for the estimation, increased public disclosure of vulnerabilities was found to escalate the frequency of data breaches, whereas cross-border data flow decreased the number of data breaches. Furthermore, the mechanism by which academic strength affects data breaches was examined through the two mediators of cross-border data flow and vulnerability disclosure. In addition, cloud adoption reduced data breaches, and public clouds were determined to be relatively more secure than private clouds. Cloud adoption also acted as a moderator between the negative impact of vulnerabilities and the positive impact of cross-border data flow on data breaches. The estimation and robustness findings revealed the underlying mechanisms that impacted university data security, clarifying the understanding of data breaches and suggesting practical implications for universities and other institutes to improve information security. The findings of this study provide insights and directions for future research.

## Introduction

**Fig. 1:**
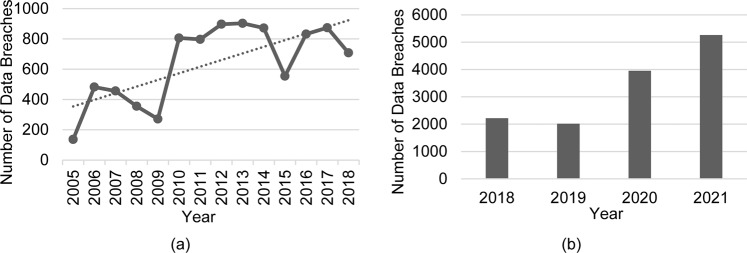
Yearly number of data breaches. A description of annual number of data breach incidents. Panel **a** presents statistics using data from PRC and panel **b** describes data breaches in recent years using data from Verizon.

Given advancements in the digital economy worldwide and the rapid development of related technologies, such as 5G and artificial intelligence, data have become an important resource globally. However, numerous potential risks of data breaches accompany such developments in information technology (IT). It has been reported that more than 100 million Android users’ sensitive personal data were exposed in May 2021 because of several misconfigurations. In the same year, a database containing the personal information of 533 million Facebook account users across 106 countries was exposed, potentially leading to further social engineering attacks or hacking attempts (Henriquez, 2021). The frequent incidents reported in the media reflect the severity of these data breaches and merely represent the “tip of the iceberg.” Despite the related laws and data breach notification requirements enacted by governments worldwide, such as the *General Data Protection Regulation (GDPR)* of the European Union, *National Security and Personal Data Protection Act of 2019 (NSPDPA)* of the United States, and *Data Security Law* and *Personal Information Protection Law* of China, data breach incidents continue to occur. Statista reported that the annual number of data compromises has increased from 2005 to 2022^1^. According to statistics from the Privacy Rights Clearinghouse, the occurrence of data breaches has been high since 2010^2^. As shown in Fig. 1(a), from 2005 to 2018, the number of reported data breach incidents increased by 4.2 times, presenting a significant upward trend. Figure 1(b) indicates that the number of data breaches achieved a record high in 2021 (Verizon, 2022). The cost of a data breach has also increased significantly. The average total cost of a global data breach was $4.35 million in 2022, which was the highest in the history of the report, increasing by 2.6% from 2021 and 12.7% from 2020. Given the COVID-19 outbreak enforcing remote work and digital transformations in recent years, data breach costs increased by $1.07 million in 2021 and $0.97 million in 2022 (IBM, 2021, 2022). Moreover, Meng et al. (2022) suggested that the spreading online of public opinions can have severe consequences. Information breaches once disclosed may damage the image of the related organization, industry, or even the supervisors.

Data breach risks remain prevalent in universities. The number of reported data breach incidents in higher education institutions is increasing (Bongiovanni, 2019). From the perspective of a university concerning multiple stakeholders (Borgman, 2018), individuals exhibit diverse activities in both physical and cyber spaces (Li T, Li Y, Hoque MA (2022) and interact through the internet, thereby leaving digital footprints (Qin et al., 2022). Students, faculty, staff, and visitors frequently access a university’s information technology infrastructure and generate data in various ways, such as via personal mobile devices, laboratory sensors, and swipe card access systems. These large-scale data interactions and flows among organizations and users inadvertently and continuously expand the digital footprints of universities, potentially leading to information security concerns by increasing the risk of data breaches. Moreover, insufficient security awareness and a lack of attention to data security place universities in a dangerous position. According to a survey by the Joint Information Systems Committee (JISC), only 39% of students indicated that they were informed of how universities store and use their personal data. Only 15% of the staff scored their organizations as eight or more out of ten in terms of data protection (JISC, 2018). Notably, JISC had a 100% track record of gaining access to the most valuable data in universities and research centers using spear phishing (Chapman, 2019). Data breaches may also be caused by human errors, such as sloppy data handling and negligent security procedures, due to insufficient awareness of data security (Ulven and Wangen, 2021). For example, almost 44,000 student records were obtained from the storage of secure information at Arden University in 2022 because of human errors^3^. Moreover, according to Verizon (2022), the education sector has been facing additional challenges because the pandemic made it mandatory to hold classes online, providing opportunities for malicious hackers and increasing the risk of data breaches.

Universities with plentiful personal and research data, intellectual property, and insufficient awareness of data security are enticing from a hacker’s perspective, making higher educational institutions primary targets (Hina and Dominic, 2020). It has been observed that the number of information security breach incidents reported by higher education institutions worldwide is increasing rapidly (Borgman, 2018). For example, the University of California announced a malicious cyberattack in 2021, and the stolen personal information (e.g., social security numbers, email addresses, phone numbers, and home addresses) was found on the dark web (Ying, 2021).

**Fig. 2:**
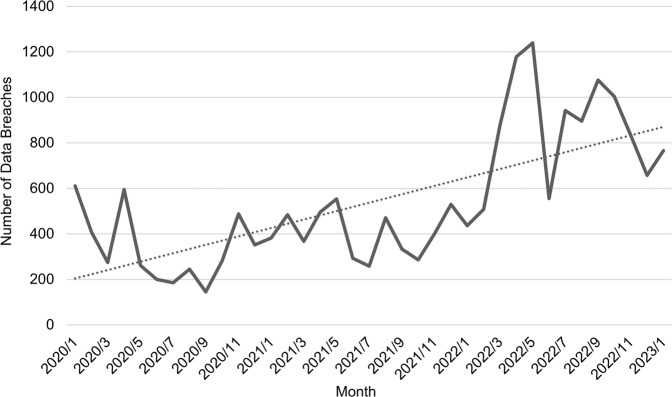
Monthly statistics of data breaches in universities. A description of data breach incidents in universities in China using data from the Education Industry Vulnerability Reporting Platform.

The same holds true for data breach risks in universities in China. Figure 2 presents the monthly statistics regarding data breach incidents in universities in China as reported by the Education Industry Vulnerability Reporting Platform, a resource-sharing platform for collection and notification of system vulnerabilities in the country’s education industry^4^. The number of reported data breaches was relatively high, with a significant upward trend on a monthly basis, reflecting that Chinese universities are also at severe risk of data breaches, which should not be underestimated.

Despite the increasing trend in information breach incidents, previous studies have rarely focused on such incidents in universities (Okibo and Ochiche, 2014). As Hina and Dominic (2020) have reported, only a few studies have focused on the security risks of sensitive information from higher educational institutions. Information security management in universities is a poorly investigated topic (Bongiovanni, 2019).

Hence, in this study, which focuses on universities’ data breach incidents, we aim to investigate the determinants of data breach risks to better understand the underlying impact mechanisms. The research framework is at the university level, and the samples used in empirical analyses were obtained from China. The aim of this study is to answer the following research questions: (i) What factors impact data breaches in universities and how do these factors interact? (ii) By what mechanism does academic strength impact data breaches? (iii) What is the influence of emerging information technologies, such as cloud storage, on the impact mechanism?

Based on crime opportunity theory and routine activity theory, we examine how public vulnerability disclosures, cross-border data flow, academic strength, and the adoption of cloud storage affect the possibility of data breaches, thereby analyzing the interactions between these variables. It is observed that an increase in the number of public disclosures of vulnerabilities increases the frequency of data breaches. In addition, cross-border data flow decreases the number of data breaches. Subsequently, using two mediators, the mechanisms through which academic strength affects data breaches are identified. Universities with higher academic achievements have relatively higher cross-border data flow and vulnerabilities. Furthermore, cloud storage is better than local storage when considering data breaches, and a public cloud has better performance than a private cloud in data security protection. Furthermore, our study shows that cloud adoption negatively moderates the impacts of vulnerabilities and positively moderates cross-border data flows.

This study contributes to the literature in several ways. First, the factors influencing data breach incidents related to universities are empirically examined. Prior studies focusing on data breach risks have primarily considered the medical industry and enterprises. The higher education industry—particularly universities—is presently subject to severe data breach risks, but has received relatively limited attention. Second, as risk management has become a research focus in the context of cross-border data flow, we investigate the effects of cross-border data flow on data breaches and provide a new perspective for understanding the value of such data transfers. Third, the impact of the cloud on data breaches is identified, distinguishing between the effects of different types of cloud adoption. Finally, we contribute to the literature on data breaches and theories on data security, indicating several managerial implications for the control of data security risks in universities to further optimize data protection strategies.

The remainder of this paper is organized as follows. First, the relevant literature is reviewed. Second, related theories are outlined prior to proposing a research framework with hypotheses. Third, the data and variable measurements are described, followed by empirical analyses and main results. Subsequently, several robustness checks are performed. Finally, the results are discussed, and conclusions are drawn.

## Literature review

Prior research has analyzed the motives behind cybersecurity and the influencing factors of data breaches. Factors such as organizational attributes, economic indicators, and information technologies have been empirically explored. In this section, we first review the literature related to information breaches in universities and then summarize the literature according to types of influencing factors.

### Information breaches in universities

As demonstrated by Bongiovanni (2019), regarding security management, information in universities is the least secure. Data breaches in higher education are becoming increasingly common (Chapman, 2019). One of the most urgent threats faced by higher education is from cybercriminals or hackers seeking to profit from the theft of the sensitive personal and financial information of the students, faculty, and staff (FireEye, 2016). Verizon (2022) noted that monetary gain was the primary motive for approximately 95% of data breach incidents observed in higher education in 2021. In general, the intention of cybercriminals is to steal data that can be quickly monetized.

The open and collaborative environment in a university and the typical access to many portable devices make it easier to gain access to unauthorized sensitive information (Coleman and Purcell, 2015). Web users are highly mobile and accustomed to accessing the web from any device, at any time, and from anywhere. This open-design architecture commonly used by universities undoubtedly facilitates the exchange of information (Okibo and Ochiche, 2014); however, the existence of numerous connected devices across organizations, the coexistence of different security cultures, and the tendency to outsource security controls make universities more vulnerable to information security risks (Borgman, 2018). Additionally, the academic culture of openness and the unencumbered access make it particularly difficult for universities to maintain security. The lax security that facilitates open access and the sharing of cutting-edge academic research and content on the network makes higher education an attractive target for attackers (Roman, 2014). In conclusion, universities that hold sensitive personal data and intellectual property of many researchers are ideal targets (Chabrow, 2015).

One of the factors affecting information security in universities is the increasing difficulty of security management. Noghondar et al. (2012) pointed out that high turnover rates and general complacency toward information security also increase the exposure of university information. Magura et al. (2021) highlighted issues affecting database security that could lead to data breaches and data theft, including human factors, work environments, and the technologies used. Liu et al. (2020) studied how centralized IT decision-making affects the likelihood of cybersecurity breaches in higher education, especially in institutions with a more heterogeneous IT infrastructure. Iriqat et al. (2019) explored the compliance of staff with information security policies at the Palestine University. Other studies have concluded that a lack of security awareness is directly related to how the faculty value the information system assets of their universities (Nyblom et al., 2020). To address these concerns, artificial neural network techniques have been utilized to improve cybersecurity in higher education (Saad AL-Malaise AL-Ghamdi et al., 2022).

### Data breach influencing factors

There are three typical types of research on data breaches: (1) *analysis of the consequences of data breaches*, such as that of Foerderer and Schuetz (2022), who studied the influence on stock market reactions, Ali et al. (2022), who focused on the long-term effects on equity risk, and Bachura et al. (2022), who investigated the emotional response after a data breach and identified breach concepts most relevant to each emotion; (2) *research on response strategies*, such as user compensation (Goode et al., 2017; Hoehle et al., 2022) and corrective action (Nikkhah and Grover, 2022); and (3) *analysis of the causes of data breaches*, which we focus on primarily in this paper. The most relevant existing studies on the influencing factors of data breaches from different sectors listed in Table 1 provide a comparative analysis primarily from an industry perspective. From the listed studies, it can be concluded that when organizations at risk of data breaches have more commercial attributes, the interests involved can be more complex; thus, social perception can significantly affect information security, especially the likelihood of cyberattacks. However, when an organization has fewer commercial attributes, the defining attributes of the organization and IT management are dominant factors that influence data security. In the case of companies, due to their special nature as business organizations, researchers are more concerned about the impact of a company’s performance and image, which is likely to cause dissatisfaction among stakeholders (D’Arcy et al., 2020). In addition, the management practices of employees and the personal characteristics of top managers are important factors related to information security (Ifinedo, 2016; Haislip et al., 2021; Burns et al., 2022). Studies related to the health care industry have largely focused on organizational features. Scholars have paid more attention to the impact of IT management systems and organizational characteristics on data security (Angst et al., 2017; Dolezel and McLeod, 2019; Kim and Kwon, 2019). The same holds true for higher education, especially for Chinese universities as they are generally public universities with fewer commercial features. Therefore, following the spirit of prior research, this study focused on organizational features and IT measures.

**Table 1:** Competitive analysis.

Studies	Sector	Dependent variable(s)	Independent variable(s)
Sen and Borle (2015)	/	Data breach risks	Vulnerabilities, IT security investment, economic indicators, data breach disclosure laws
Liu et al. (2020)	Higher Education	Cybersecurity breaches	IT Centralization, IT Heterogeneity
D’Arcy et al. (2020)	Corporate	Computer attacks	Social performance
Ifinedo (2016)	Corporate	Employee compliance	Top management support, severity of sanctions, cost-benefit analyses
Burns et al. (2022)	Corporate	Insider computer abuse	Personal motives and controls
Haislip et al. (2021)	Corporate	Data security risks	Executives’ IT expertize
Dolezel and McLeod (2019)	Healthcare	Data security risks	Employee behavior, safety culture, training, supplier selection, risk management procedures
Kim and Kwon (2019)	Healthcare	Accidental and malicious data breaches	Electronic medical record, medical management department plan
Angst et al. (2017)	Healthcare	Data breaches	Integration of security, IT-related processes
Pang and Tanriverdi (2022)	Federal agencies	Cybersecurity risks	Cloud migration of legacy IT systems

The risks of data breaches can differ based on the main industry, geographic location, and types of breaches occurring in the past (Sen and Borle, 2015). Lee and Hess (2022) found that demographic variables (gender, age, race, ethnicity, income, and location) and political ideology are associated with data security. Schlackl et al. (2022) summarized the antecedents of data breaches identified in prior research, including technology measures, information disclosure, organization attributes, etc. In an enterprise, corporate social performance (measured by participation in socially responsible or irresponsible activities) has been proven to affect the likelihood of computer attacks leading to data breaches (D’Arcy et al., 2020). Corporate reputations were found to be important assets in protecting corporate value after a data breach (Gwebu et al., 2020). Wang and Ngai (2022) explored the negative association between firm diversity and data breach risks, delineating the boundary conditions. Ifinedo (2016) discussed how top management support, the severity of sanctions, and cost‒benefit analyses have significantly impacted employee compliance with information systems security policies. Burns et al. (2022) studied personal motives and controls for insider computer abuse, which could lead to costly and severe data breaches. Regarding the medical industry, Wasserman and Wasserman (2022) focused on cybersecurity risks in hospitals. Dolezel and McLeod (2019) studied employee behavior, safety culture, training, supplier selection and handling of personal health information, and strong risk management procedures as data breach factors. Another study found that data breach risks differ according to type and scale of a hospital (Gabriel et al., 2018). Regarding the banking industry, Ali et al. (2020) investigated the effects of socio-factors on the banking sector’s systematic risks.

Given the emerging developments of new information technologies, such as artificial intelligence and intelligent robots (Ban et al., 2022; Lu et al., 2023), IT factors are attracting more attention in related research streams. IT investments have been found to be effective in reducing the risk of data breaches (Sen and Borle, 2015); however, this does not necessarily translate into fewer data breaches. Institutional factors create conditions under which IT security investments can perform more effectively. When considering the impact of information security investments on data breaches, companies must consider the impact of institutional factors and balance them. Li et al. (2021) found that IT security investments have different effects on security breaches in organizations with different approaches to making digitalized progress. Li W, Leung ACM, Yue WT (2022) stated that there is a dynamic interrelationship between IT investments and data breaches. Haislip et al. (2021) found that executives’ IT expertize could be an effective factor influencing reported data security breaches. Additionally, the increase in vulnerabilities adds to the risk of data breaches but is mitigated by an increase in expired vulnerabilities (Sen and Borle, 2015). Regarding new ITs, Fried (1994) discussed both new threats and potential new defenses for information systems security brought about by new products and information technologies. For example, Kim and Kwon (2019) found that electronic medical records and medical management department plans increase the risk of accidental and malicious data breaches, especially in larger hospitals. For emerging cloud services, although people generally believe that cloud services are more vulnerable to security breaches, cloud services in fact reduce the average expected losses of consumers relative to internal software in a high-security loss environment during an attack (Zhang et al., 2020). Moreover, cloud storage is a type of centralized storage (Bandara et al., 2021; Ouf and Nasr, 2015; Wu et al., 2014) and may be safer when considering the emergence of end-user computing. The task of ensuring information security becomes more complex as information systems become increasingly distributed (Fried, 1993), and the integration of security and IT-related processes can reduce data breaches (Angst et al., 2017). Pang and Tanriverdi (2022) found that cloud migration of legacy IT systems significantly reduces cybersecurity risks for public clouds through the internal and external guardianship provided by the cloud service, which has more resources for establishing effective information protection.

As the digital economy develops, additional discussions on the security and development of cross-border data flow have emerged. The benefits of cross-border data have both economic and social repercussions. Ten percent of the average profit growth of various industries is attributed to cross-border data (China Academy of Information and Communications Technology, 2021). Bauer et al. (2013) found that limiting the free flow of data leads to a reduction in gross domestic product (GDP). In terms of social benefits and public welfare value, the Organization for Economic Co-operation and Development (OECD) (2019) insists that it is necessary for data to flow domestically and internationally, as this can provide significant developmental benefits. The “public good” nature of data beyond national borders has been emphasized and calls for international data sharing. For example, the COVID-19 pandemic clearly demonstrated the importance of the global sharing of health data for research purposes (United Nations Conference on Trade and Development, 2021). However, cross-border data flow and international storage are associated with perceived risks, such as those concerning surveillance and unwarranted data mining (Meltzer, 2015). To assess risk, Li et al. (2022) developed a risk index system for cross-border data flow and applied it to biomedical organizations. There is evidence that localized data are unlikely to provide better results in terms of data breaches, and the domestic storage of data poses risks to many poorly managed and costly data centers (Chander and Lê, 2014). Indeed, data localization does not contribute to data security but makes it more vulnerable to destruction, especially by hackers (Chander and Lê, 2015).

In summary, the current literature has shown that great progress has been made in research on the factors that influence data breaches, thereby drawing a basic outline of the problem and providing a thorough comprehension of data breaches. Based on this, we focus on identifying the influencing factors related to universities.

## Theories and hypotheses

### Relevant theories

The *routine activity theory* proposes three factors leading to crimes (in this case, cybersecurity crimes): (i) potential attackers or malicious insiders with crime motives; (ii) suitable, accessible, and valuable targets; and (iii) a lack of competent guardianship (Cohen and Felson, 1979). In this context, offenders can be predominantly potential attackers, malicious insiders, or insiders who disclose sensitive information unintentionally (Pang and Tanriverdi, 2022). The motive is mainly financial (Verizon, 2022). The target could be accessible IT systems that manage universities’ critical information. Universities can strengthen their guardianship by investing in security protection technology (Liao et al., 2017; Luo et al., 2020; Wang et al., 2015) or by seeking external governance from vendors (Pang and Tanriverdi, 2022).

The central assumption of *crime opportunity theory* is that criminal behavior is driven by human rationality and that the conditions for committing a crime require a vulnerable victim in addition to motive and the lack of restraint (Hannon, 2002). Thus, criminals are more likely to take opportunistic actions and choose victims who are more vulnerable. In criminal cases that lead to data breaches, vulnerabilities in information systems, software, and firmware present opportunities for potential intruders, that is, the more system vulnerabilities there are, the greater the chances of attracting intruders will be, resulting in a higher risk of data breaches.

### Hypotheses development

**Fig. 3:**
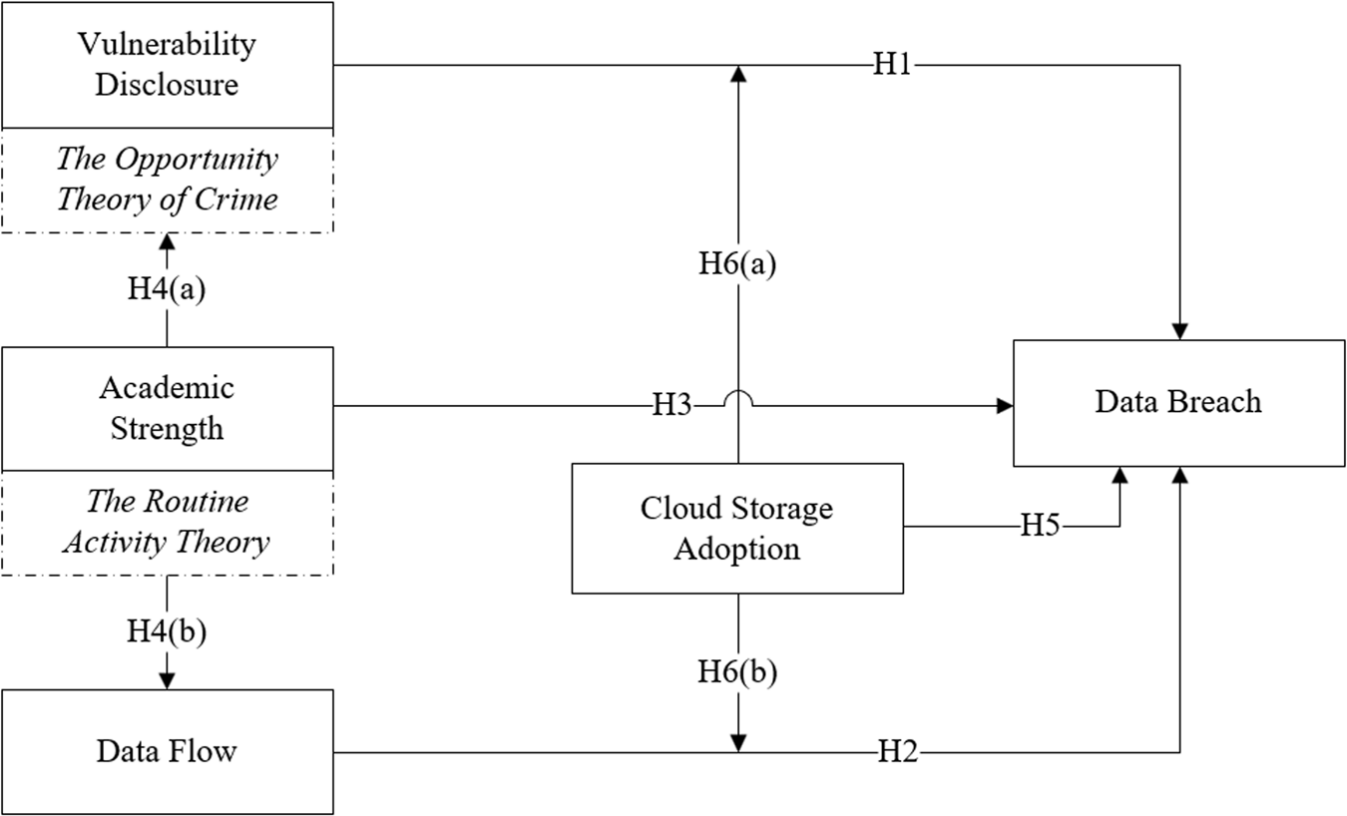
Research framework. It shows the relationships among variables and demonstrates relevant theories applied.

Based on relevant theories and the related literature, we propose the research framework shown in Fig. 3 and the following research hypotheses.

Coordinated vulnerability disclosure (CVD) is an efficient approach to finding and fixing flaws in IT systems. Through this approach, after finding a vulnerability in an IT system, a white-hat hacker (an ethical hacker who uses his or her ability to discover security vulnerabilities and helps protect organizations) reports it to the platform to warn the system manager. Details such as the titles of the vulnerabilities and their brief descriptions, ratings, and comments are visible to all registered white hats. However, the vulnerability details are only visible to relevant organizational administrators and vulnerability submitters. According to *crime opportunity theory*, criminals are more likely to engage in speculative behavior and choose victims who are more vulnerable (Hannon, 2002). In data breach incidents, “vulnerable” represents the public disclosure of computer security vulnerabilities in information systems, software, and firmware (Sen and Borle, 2015), which enhances the accessibility of sensitive information, thereby increasing the data breach risk according to *routine activity theory*. It has also been noted in the literature that public disclosures of relevant vulnerabilities increase the frequency of attacks (Browne et al., 2001). The more vulnerabilities there are, the more vulnerable the information system is to malicious attackers. Therefore, we propose the following hypothesis.

H1: Public disclosure of vulnerabilities increases data breaches.

Given the development of globalization, cross-border data flow has become an essential part of the global digital economy. The necessity for cross-border data flow has been emphasized considering its significant economic and social benefits (Bauer et al., 2013; OECD, 2019), especially in the context of academic research on international collaborations and data exchanges. It is also evident that localizing data storage is unlikely to provide better results in terms of data breaches (Chander and Lê, 2014) and does not contribute to data security; instead, it makes the data more vulnerable to destruction, especially by hackers (Chander and Lê, 2015). Rather than reducing data security risks, suppressing cross-border data flow places universities at a disadvantage. Thus, universities with greater cross-border data flow may have fewer data breach incidents. Therefore, we propose the following hypothesis.

H2: There is a negative relationship between the frequency of cross-border data flow and occurrences of data breaches.

According to the Data Breach Investigation Report by Verizon (2022), more than 75% of breach incidents in the education industry are by external attackers. Financial motives account for 95% of attacker motives, meaning that hackers mostly attack for money (e.g., by selling personal information and through blackmail). Academics are the heart of a university, and the performance of the faculty affects the quality of student learning and the strength of the university, which in turn impacts the contributions of academic institutions to society (Shrand and Ronnie, 2019). Many indicators of research success are significantly associated with a university’s reputation (Linton et al., 2011). In higher-ranked universities, the volume of research is larger. According to *routine activity theory*, offenders tend to choose more valuable targets. Therefore, hackers who hack for money are more likely to aim for academically stronger universities, as they are more famous and perform better in industry. Similar concerns have been raised in previous research. Liu et al. (2020) considered the impact of research grants on cybersecurity attacks since the valuable intellectual properties generated in research and development activities are at risk of being stolen and misappropriated, which makes universities particularly attractive targets for cybersecurity attacks. In other words, academically stronger universities are more likely to be attacked, leading to additional data breaches. Hence, we propose the following hypothesis.

H3: There is a positive relationship between academic strength and the number of data breaches.

According to Weulen Kranenbarg et al. (2018), one motive for white-hat hackers’ CVD reporting is to gain status in the hacker community, as they expect recognition and acknowledgment. The other motive is cash bounties, which account for 15% of motives. However, considering that no such bounty programs exist on the Education Industry Vulnerability Reporting Platform and that only gifts can be redeemed, we assume that the main motivations for CVDs by hackers are to gain status regarding and acknowledgment of their skills and actions. Undoubtedly, CVDs are aimed at more famous and influential universities, in contrast to “normal” universities. Therefore, the vulnerabilities of universities with higher academic achievements and greater social impact are more likely to be reported or disclosed to attract more social attention. Based on this, we propose that such vulnerabilities mediate the relationship between academic strength and data breaches.

H4(a): The number of vulnerabilities has a mediating effect on the relationship between research strength and the number of breaches.

Considering that universities with stronger academic strength have broader worldwide influence and more academic communication with foreign institutions and individuals, they may commit to larger-scale, global data flow around the world. Therefore, we propose that the scale of cross-border data flow mediates the relationship between academic strength and data breaches.

H4(b): The amount of cross-border data flow has a mediating effect on the relationship between research strength and the number of breaches.

Millions of companies and institutions use the cloud to store data remotely and run applications and services, thereby reducing costs and accelerating operations (Rawding and Sacks, 2020). According to the Cloud Usage and Digital Economy Development Report (2018) of the Tencent Research Institute, the degree of “cloudification” is an important indicator of digital economy development. Zhang et al. (2020) investigated the security risks posed by cloud computing services and found that cloud adoption can significantly reduce losses from data breaches. Pang and Tanriverdi (2022) concluded that performing cloud migration for legacy IT systems significantly reduced cybersecurity risks. It has been shown that maintaining information security in a distributed environment brings challenges to information security management (Fried, 1993) and that the integration of IT-related processes can further reduce data breaches (Angst et al., 2017). Therefore, cloud storage, as a type of centralized storage (Bandara et al., 2021; Ouf and Nasr, 2015; Wu et al., 2014), may be the safer option for avoiding data breaches. Thus, we propose the following hypothesis.

H5: Universities adopting cloud storage present a relatively lower frequency of data breaches.

Cloud storage could be safer because of the centralization and integration of related processes, making maintenance and management easier and resulting in fewer vulnerabilities. Therefore, cloud storage has a moderating effect on the relationship between the number of disclosed vulnerabilities and the number of data breaches. When cloud storage is adopted, there will be fewer vulnerabilities and thus fewer data breaches. Thus, we propose the following hypothesis.

H6(a): Adopting cloud storage plays a negative moderating role in the relationship between the number of vulnerabilities and the number of data breaches.

Moreover, cloud computing enables a larger volume of data from across the world to flow over a larger area. Cloud services accessed remotely over the internet can serve customers across national boundaries and achieve cross-border data flow, which enables reduced unit costs and prices over time and flexible and technologically sophisticated services (Coyle and Nguyen, 2019). Thus, cloud storage makes it easier to achieve data transmission without geographical restrictions/changes; accordingly, it may promote data flow in universities. Therefore, cloud storage has a positive moderating effect on the relationship between the number of cross-border data flows and data breaches. When cloud storage is adopted, there is greater cross-border data flow and thus fewer data breaches. Hence, we propose the following hypothesis.

H6(b): Adopting cloud storage plays a positive moderating role in the relationship between the number of cross-border data flows and the number of data breaches.

## Data and variables

### Sample data collection

The data used in this study were drawn from Chinese universities, where data breaches pose a risk to personal privacy, intellectual property rights, and even national security, especially during the COVID-19 epidemic when most courses were delivered online.

**Fig. 4:**
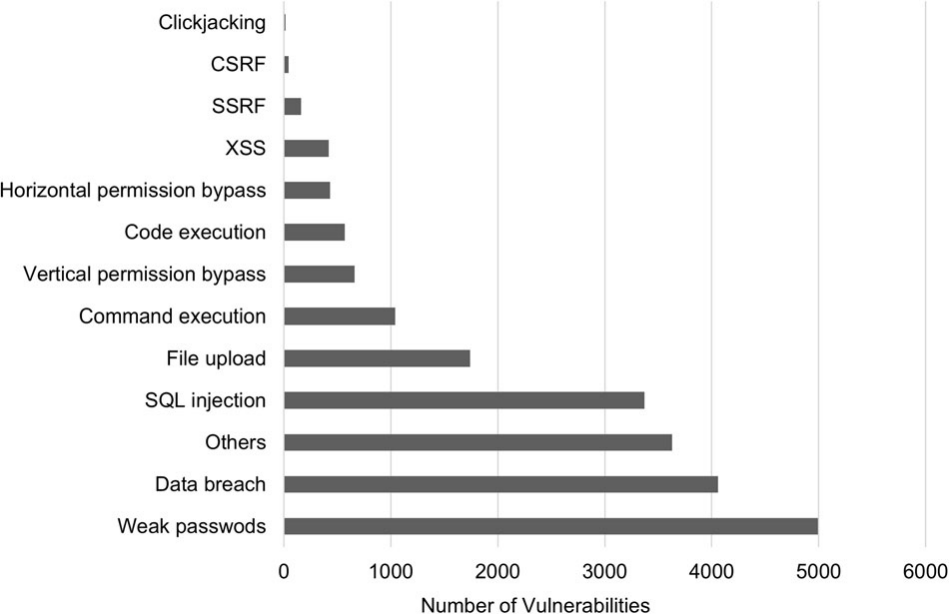
Numbers of different types of vulnerabilities. A description of the types of vulnerabilities and the distribution of each type of vulnerability using data from the Education Industry Vulnerability Reporting Platform.

The study samples were collected as follows. First, 21,135 records from January 1, 2020, to January 1, 2021, related to breach incidents and public disclosures of vulnerabilities were collected from the Education Industry Vulnerability Reporting Platform (https://src.sjtu.edu.cn/). After removing the data for education departments of provincial governments and junior colleges, a dataset comprising 681 universities with a total of 9916 records was obtained. Each record contained information on the university/institution name, author, event description, vulnerability type, and risk level. The types of vulnerabilities were classified as sensitive information breaches, structured query language injection vulnerabilities, vertical/horizontal permission bypasses, weak passwords, or file upload vulnerabilities. The statistical results of the vulnerability types are shown in Fig. 4. Breaches of sensitive information accounted for more than 30% of all vulnerabilities. Except for breaches, other incidents were mainly caused by technical failures.

Second, we obtained records of cross-border data flow from the experimental logs of collaborating institutions. By excluding data of research institutes and enterprises, we obtained valid records on the cross-border data of 110 universities, which are included in the list of 681 already collected.

Third, we collected other university-related information using different methods based on the list of 110 universities. Among them, data on research projects for measuring research strength were collected from the LetPub Fund Project Inquiry System (http://www.letpub.com.cn/); university-related information for measuring control variables and cloud adoption were collected from universities’ official homepage sites; and economic statistics were collected from the website of the National Bureau of Statistics.

Finally, after matching the data, a valid dataset comprising 110 universities and more than 900 valid data breach records was obtained for further analysis.

### Variables and measurements

The number of data breaches in universities in 2020 was used as the dependent variable to reflect universities’ data security status. For the independent variables, the number of data transmissions abroad was used as a measure of cross-border data flow. Following a prior study (Sen and Borle, 2015), the number of publicly reported vulnerabilities was used to measure vulnerability disclosure. The number of research projects was used to measure the academic strengths of the universities. Cloud storage utilization was coded “1” if a university had cloud storage at the beginning of 2020 and “0” otherwise.

According to Gartner, using contextual information, such as location and time data, can help users understand dynamic information security environments and make more accurate security decision^5^. According to Say and Vasudeva (2020), experiencing a failure can expose an organization’s potential problems and weaknesses, whereby the organization acquires important experience and lessons to reduce the possibility of subsequent failures. For universities aiming to prevent future breach incidents, training in information security is one of the best remedial measures. Therefore, in this study, to capture the differences caused by training, we controlled for the relevant IT security training conducted at universities. It has also been empirically shown that institutions’ scales are positively related to the risk of data breaches (Gabriel et al., 2018; Kim and Kwon, 2019). Therefore, the scale of the universities was controlled, as demonstrated by the number of undergraduate majors. In addition, economic indicators were found to be positively correlated with the risk of data breaches (Sen and Borle, 2015); thus, we also controlled for the GDP of the city where each university was located. In addition, other control variables were added for the number of national key disciplines, master’s programs, doctoral programs, time of establishment, type of university, attributes of university, and number of universities in the same city. The detailed definitions of the variables are provided in Table 2.

**Table 2:** Definitions of variables.

Variable name	Definition	Measurement	Reference
*Num_Breach*	Data breach risk	Number of data breaches	D’arcy et al. (2020); Kim and Kwon (2019);
*Num_Data_Flow*	Cross-border data flow	Number of cross-border data flows (unit: ten thousand times)	Li et al. (2022)
*Num_Vulnerability*	Disclosure of vulnerabilities	Number of system vulnerabilities	Sen and Borle (2015); Browne et al. (2001)
*Ind_Cloud_Storage*	Cloud storage adoption	Whether the cloud is used to store data, yes = 1, no = 0	Pang and Tanriverdi (2022); Zhang et al. (2020)
*Num_Research_Project*	Academic strength	Number of research projects (unit: one hundred)	Liu et al. (2020)
*Ind_Training*	Relevant training	Whether relevant training is conducted, yes = 1, no = 0	Say & Vasudeva (2020); Dolezel and McLeod (2019)
*Num_Undergraduate_Major*	Scale of university	Number of undergraduate majors	Liu et al. (2020)
*Num_Master_Program*	Capability for master training	Number of Master programs	Liu et al. (2020)
*Num_Doctoral_Program*	Capability for doctor training	Number of Doctoral programs	Liu et al. (2020)
*Num_Key_Disciplines*	Comprehensive strength	Number of key disciplines	Liu et al. (2020)
*Time_Duration*	Established time	Established time till 2020	Kim and Kwon (2019);
*Ind_Univ_Type*	Type of university	Whether the university is a comprehensive one, yes = 1, no = 0	Gabriel et al. (2018); Wang (2022)
*Att_Univ*	Attribute of university	Attribute of the university, i.e., 985, 211, and others	Gabriel et al. (2018); Wang (2022)
*City_GDP*	Economic development indicators	GDP of the city in 2019 (unit: 107 billion yuan)	Lee and Hess (2022); Wang (2022)
*Num_Univ_City*	Educational development indicators	Number of universities in the city where the university is located	Bloom et al. (2015); Liu et al. (2020)

### Descriptive statistics

Table 3 describes the statistics calculated for the main variables. Although the data security risks of universities appeared uneven, they generally faced a severe risk of data breaches, with a mean value of 8.5 breaches in 2020. Among the 110 universities, in terms of attributes (only the highest title of the university was taken), 20% were universities in “Project 985” and 35% were universities in “Project 211”^6^. In addition, 48% of the universities were comprehensive universities, whereas 52% were noncomprehensive universities (such as those limited to medicine, finance and economics, normal education, or science and engineering). Regarding the urban distribution of universities, 23% were in the most developed first-tier cities; 40% were located in new first-tier cities^7^; and the rest were from less-developed areas.

**Table 3:** Descriptive statistics.

Variable	Obs.	Mean	S.D.	Min	Max
*Num_Breach*	110	8.464	10.88	0.0	89.0
*Num_Data_Flow*	110	2.176	7.161	0.0	48.4
*Num_Vulnerability*	110	31.48	62.17	0.0	605
*Num_Research_Project*	110	73.86	31.22	16	139
*Ind_Cloud_Storage*	110	0.336	0.475	0.0	1.00
*Num_Undergraduate_Major*	110	250.3	379.7	1.0	2380
*Ind_Training*	110	0.973	0.164	0.0	1.00
*Num_Master_Program*	110	76.31	76.27	6.0	443
*Num_Doctoral_Program*	110	32.22	47.07	1.0	334
*Num_Key_Disciplines*	110	7.618	15.14	0.0	95.0
*Time_Duration*	110	13.81	13.40	0	60
*City_GDP*	110	1.486	1.087	0.054	3.816
*Num_Univ_City*	110	49.22	25.30	1	97

Table 4 presents the correlation matrix. Considering that some correlations were high, and that multicollinearity may have existed among the variables, we conducted a variance inflation factor (VIF) test. Except for the largest VIF value of 3.39 (*Num_Research_Project*), the remaining VIF values were no higher than 3, indicating no significant multicollinearity issues.

**Table 4:** (**a**) Correlation matrix. (**b**) Correlation matrix.

(a) Variable	(0)	(1)	(2)	(3)	(4)	(5)	(6)
*Num_Breach*	1						
*Num_Data_Flow*	**0.49**	1					
*Num_Vulnerability*	**0.90**	**0.53**	1				
*Num_Research_Project*	**0.61**	**0.73**	**0.59**	1			
*Ind_Cloud_Storage*	0.08	0.05	0.11	0.12	1		
*Ind_Training*	0.03	−0.10	0.07	0.03	0.001	1	
*Num_Undergraduate_Major*	**0.34**	0.11	0.17	0.24	0.028	**0.25**	1
*Num_Master_Program*	0.24	−0.02	0.12	0.24	−0.10	0.11	**0.38**
*Num_Doctoral_Program*	0.18	0.11	0.14	0.39	−0.03	0.08	**0.31**
*Num_Key_Disciplines*	**0.38**	**0.26**	**0.27**	**0.46**	**0.25**	0.10	**0.33**
*City_GDP*	**0.27**	**0.34**	**0.27**	**0.33**	0.21	−0.08	−0.13
*Num_Univ_City*	0.10	0.10	0.12	0.23	0.19	0.09	−0.11
*Time_Duration*	**0.32**	0.16	**0.26**	**0.30**	0.12	0.24	**0.34**

(b) Variable	(7)	(8)	(9)	(10)	(11)	(12)
*(7) Num_Master_Program*	1					
*(8) Num_Doctoral_Program*	**0.72**	1				
*(9) Num_Key_Disciplines*	**0.35**	0.41	1			
*(10) City_GDP*	−0.10	0.03	0.23	1		
*(11) Num_Univ_City*	−0.03	0.16	**0.30**	**0.66**	1	
*(12) Time_Duration*	0.20	0.24	**0.40**	0.10	0.22	1

Bolded values are significant at the *p* < 0.05 level.

## Empirical analyses

### Main results

To empirically test the proposed hypotheses, we constructed the following baseline model for analysis.

1 $$\begin{array}{l}\log \left( {E\left( {Num\_Breach_i\left| {X_i} \right. + Controls} \right)} \right)\\ \quad = \alpha + \beta _1Num\_Vulnerability_i + \beta _2Num\_Research\_Project_i\\ \qquad+ \,\beta _3Num\_Data\_Flow_i + \beta _4Ind \_Cloud\_Storage_i + \gamma Controls\end{array}$$

Poisson regression was employed since the dependent variable *Num_Breach* was measured using discrete and countable data, representing the number of reported data breaches for university *i*; *X*_*i*_ is a vector of the independent variables; *Num_Vulnerability*_*i*_ is the total number of disclosed vulnerabilities of university *i*; *Num_Research_Project*_*i*_ is the number of research projects of university *i*; *Num_Data_Flow*_*i*_ is the number of cross-border data flows of university *i* in 2020; *Ind_Cloud_Storage*_*i*_ indicates the data storage method of university *i*; *Controls* denotes a series of control variables, including training commitment, number of doctoral programs and master’s programs, number of national key disciplines, years since university establishment, type of the university, attributes of the university, GDP, and number of universities in the city where the university is located.

The empirical results are presented in Table 5. Column (1) presents the results for the control variables. Column (2) presents the full Poisson regression model. A multiple linear regression model and a negative binomial regression model were further run for robustness tests, and the results are presented in Columns (3) and (4), respectively. All three regressions show similar estimation results for the main variables. The full Poisson regression model displays the best goodness-of-fit among all models, with the smallest Akaike information criterion (AIC) and Bayesian information criterion (BIC) values in Column (2).

**Table 5:** Main empirical results.

Column	(1)	(2)	(3)	(4)
Regression	Poisson	Poisson	Linear	Negative Binomial
*Num_Data_Flow*		−0.021** (0.008)	−0.007 (0.013)	−0.009 (0.014)
*Num_Vulnerability*		0.005*** (0.001)	0.006*** (0.001)	0.008*** (0.001)
*Ind_Cloud_Storage*		−0.335*** (0.088)	−0.084 (0.130)	−0.115 (0.136)
*Num_Research_Project*		−0.000 (0.000)	−0.000 (0.003)	−0.000 (0.000)
*Num_Undergraduate_Major*	0.011*** (0.002)	0.013*** (0.002)	0.012*** (0.003)	0.014*** (0.003)
*Ind_Training*	−0.646* (0.254)	−0.780** (0.258)	−0.551 (0.375)	−0.716 (0.391)
*Num_Master_Program*	0.002** (0.000)	−0.001 (0.001)	0.001 (0.001)	0.001 (0.001)
*Num_Doctoral_Program*	−0.002* (0.001)	0.000 (0.001)	−0.001 (0.002)	−0.002 (0.002)
*Num_Key_Disciplines*	−0.003 (0.004)	0.017*** (0.004)	0.016 (0.009)	0.012 (0.009)
*Time_Duration*	0.007*** (0.001)	0.002 (0.002)	0.003) (0.002)	0.002 (0.003)
*Ind_Univ_Type*	−0.123 (0.101)	−0.263* (0.106)	−0.389* (0.172)	−0.335 (0.178)
*Att_Univ_985*	0.605*** (0.115)	0.271* (0.123)	0.029 (0.221)	0.162 (0.216)
*Att_Univ_211*	−0.255* (0.115)	−0.158 (0.118)	−0.168 (0.168)	−0.107 (0.177)
*City_GDP*	0.374*** (0.037)	0.222*** (0.046)	0.132 (0.079)	0.167* (0.081)
*Num_Univ_City*	−0.012*** (0.002)	−0.008*** (0.002)	−0.004 (0.003)	−0.005 (0.004)
*Sample Size*	110	110	110	110
*R*^*2*^			0.510	
*Pseudo R*^*2*^	0.362	0.507		0.082
*AIC*	857.5	675.6		676.5
*BIC*	889.9	718.8		719.7

*p < 0.05; **p < 0.01; ***p < 0.001. Pseudo *R*^2^ is McFadden’s pseudo *R*^2^ and can be explained as *R*^2^ in generalized linear models, but with a generally smaller value, as a value of 0.2–0.4 indicates an excellent model fit (Hensher and Stopher, 1979).

The results in Column (2) of Table 5 show that the public disclosure of a vulnerability has a positive and significant effect on data breaches (*Num_Vulnerability*: *β* = 0.005, *s.e*. = 0.001, *p* < 0.001), indicating that the more disclosed vulnerabilities there are, the more breach incidents occur and the greater the risks of such data breaches are. Thus, H1 is supported. The effect of the cross-border data flow on the breach is negative and significant (*Num_Data_Flow*: *β* = −0.021, *s.e*. = 0.008, *p* = 0.002), which supports H2. This shows that the higher the frequency of data flow is, the fewer reported breach incidents there are. First, data flow reflects the fluidity and mobility of data to a certain extent. In universities with strong data fluidity, data security management generally receives greater attention and thus provides a higher level of data protection. Moreover, universities with strong data flows have more open data systems. Their data security protection and high openness reduce the motivation for potential attackers. These findings provide insights for possible future research directions. For new IT utilization, the effect of cloud adoption is statistically significant (*Ind_Cloud_Storage: β* = −0.335, *s.e*. = 0.088, *p* < 0.001) and shows that universities adopting cloud storage are less likely to have breach incidents. Thus, H5 is supported. Notably, the direct effect of academic strength (proxied by *Num_Research_Project*) on data breaches is not significant, as shown in Column (2). Additional analyses and explanations are presented in the next section.

Although not the main focus of our study, the coefficients of the other control variables also merit consideration. The scale of the university, as measured by the number of undergraduate majors, increases the risk of data breaches, similar to the results of previous research (Gabriel et al., 2018). Undoubtedly, relevant training helps reduce data breaches. Noncomprehensive universities generally face more severe risks than comprehensive universities. Universities in “Project 985”, as first-tier universities in China, face a higher risk of data breaches. Interestingly, the GDP of a city has a positive effect, whereas the number of universities in the city has a negative effect. This indicates a higher risk of data breaches in developed cities and a lower risk in cities where higher education is well developed.

### Mediating effect

Contrary to our expectations, the relationship between academic strength and data breaches is not significant. In this section, we investigated the possible mediating effects of these results. First, the public disclosure of vulnerabilities was considered as a mediator. Universities with higher academic achievement and greater social impact are more likely to be reported and exposed negatively because they attract more social attention. Thus, we addressed the mediating effect of the public disclosure of vulnerabilities. The models were constructed as follows:

2 $$\begin{array}{l}Num\_Vulnerability_i = \alpha + \beta _1Num\_Research\_Project_i\\ \qquad\qquad\qquad\qquad\qquad\,\,\,+ \;\gamma Controls + \varepsilon \end{array}$$

3 $$\begin{array}{ll}\log \left( {E\left( {Num\_Breach_i\left| {X_i} \right. + Controls} \right)} \right)\\ \quad = \alpha + \beta _1Num\_Research\_Project_i + \beta _2Num\_Data\_Flow_i\\ \qquad +\, \beta _3Ind\_Cloud\_Storage_i + \gamma Controls\end{array}$$

Equation (2) verified the relationship between the number of research projects and the disclosed vulnerabilities. Equation (3) was employed to address the existence of a mediating effect based on Eq. (2). The estimation results are presented in Table 6. The first two columns show the results obtained through Eq. (2): Column (1) is for *Num_Research_Project* only, and Column (2) incorporates the related controls. Unsurprisingly, academic strength has a positive effect on the disclosure of vulnerabilities. Column (3) presents the results without *Num_Research_Project* and *Num_Vulnerability*. In Column (4), the number of research projects is positively related to the number of data breaches, without interference from the mediating variable. Column (5) replicates the main result of Column (2) in Table 5, where the effect of *Num_Research_Project* is insignificant. Therefore, we concluded that the number of research projects indirectly affects the increase in data breach incidents through the corresponding vulnerabilities, thus supporting H4(a).

**Table 6:** Mediation effect: vulnerability and academic strength.

Column	(1)	(2)	(3)	(4)	(5)
Dependent variable	*Num_Vulnerability*	*Num_Vulnerability*	*Num_Breach*	*Num_Breach*	*Num_Breach*
*Num_Data_Flow*			0.021*** (0.003)	−0.012 (0.006)	−0.021** (0.008)
*Num_Vulnerability*					0.005*** (0.001)
*Ind_Cloud_Storage*			0.031 (0.074)	−0.102 (0.079)	−0.335*** (0.088)
*Ind_Internet_Trans*			0.031 (0.074)	−0.102 (0.079)	−0.335*** (0.088)
*Num_Undergraduate_Major*		0.007 (0.236)	0.011*** (0.002)	0.009*** (0.002)	0.013*** (0.002)
*Ind_Training*		12.64 (31.34)	−0.498 (0.257)	−0.504* (0.251)	−0.780** (0.258)
***Num_Research_Project***	0.096*** (0.013)	0.097*** (0.016)		0.001*** (0.000)	−0.000 (0.000)
*Num_Master_Program*		0.120 (0.098)	0.002*** (0.000)	0.001* (0.001)	−0.001 (0.001)
*Num_Doctoral_Program*		−0.310 (0.163)	−0.002** (0.001)	−0.003*** (0.001)	0.000 (0.001)
*Num_Key_Disciplines*		−1.026 (0.724)	−0.006 (0.004)	−0.003 (0.004)	0.017*** (0.004)
*Time_Duration*		0.247 (0.208)	0.006*** (0.002)	0.004** (0.002)	0.002 (0.002)
*Ind_Univ_Type*		−7.350 (14.41)	−0.262* (0.106)	−0.337** (0.109)	−0.263* (0.106)
*Att_Univ_985*		27.10 (17.48)	0.595*** (0.116)	0.528*** (0.116)	0.271* (0.123)
*Att_Univ_211*		−12.58 (13.88)	−0.233* (0.114)	−0.305** (0.117)	−0.158 (0.118)
*City_GDP*		7.955 (6.421)	0.249*** (0.045)	0.241*** (0.045)	0.222*** (0.046)
*Num_Univ_City*		−0.366 (0.282)	−0.010*** (0.002)	−0.011*** (0.002)	−0.008*** (0.002)
*Sample Size*	110	110	110	110	110
*R*^*2*^	0.347	0.424			
*Adjusted R*^*2*^	0.341	0.366			
*Pseudo R*^*2*^			0.390	0.419	0.507
*AIC*			824.9	789.6	675.6
*BIC*			862.7	830.1	718.8

*p < 0.05; **p < 0.01; ***p < 0.001.

Similarly, we specified a model for investigating the mediating effect of the number of cross-border data flows as follows:

4 $$\begin{array}{l}Num\_Data\_Flow_i = \alpha + \beta _1Num\_Research\_Project_i\\ \qquad\qquad\qquad\qquad\quad\quad+ \gamma Controls + \varepsilon \end{array}$$

5 $$\begin{array}{l}\log \left( {E\left( {Num\_Breach_i{{{\mathrm{|}}}}X_i + Controls} \right)} \right)\\ \quad= \alpha + \beta _1Num\_Research\_Project_i + \beta _2Num\_Vulnerability_i\\ \qquad\,+ \beta _3Ind\_Cloud\_Storage_i + \gamma Controls\end{array}$$

Table 7 shows the estimation results. As expected, cross-border data flow increased with the number of research projects. Column (3) presents the results without *Num_Research_Project* and *Num_Data_Flow*. The number of research projects is positively correlated with the number of data breaches, without interference from the mediating variable, as shown in Column (4). We concluded that the number of research projects indirectly affects the increase in data breach incidents through cross-border data flow. Universities with higher academic achievements tend to communicate more with academics worldwide. Thus, H4(b) is supported.

**Table 7:** Mediation effect: cross-border data flow and academic strength.

Column	(1)	(2)	(3)	(4)	(5)
Dependent variable	*Num_Data_Flow*	*Num_Data_Flow*	*Num_Breach*	*Num_Breach*	*Num_Breach*
*Num_Data_Flow*					−0.021** (0.008)
*Num_Vulnerability*			0.004*** (0.000)	0.005*** (0.001)	0.005*** (0.001)
*Ind_Cloud_Storage*			−0.261** (0.084)	−0.291*** (0.085)	−0.335*** (0.088)
*Ind_Internet_Trans*			−0.261** (0.084)	−0.291** (0.085)	−0.335*** (0.088)
*Num_Undergraduate_Major*		0.002 (0.022)	0.012*** (0.002)	0.013** (0.002)	0.013*** (0.002)
*Ind_Training*		−3.321 (2.917)	−0.620* (0.255)	−0.693** (0.259)	−0.780** (0.258)
*Num_Research_Project*	0.014*** (0.001)	0.015*** (0.001)		−0.000** (0.000)	−0.000 (0.000)
*Num_Master_Program*		−0.013 (0.009)	0.000 (0.001)	−0.000 (0.001)	−0.001 (0.001)
*Num_Doctoral_Program*		−0.012 (0.015)	−0.001 (0.001)	0.000 (0.001)	0.000 (0.001)
*Num_Key_Disciplines*		−0.016 (0.067)	0.012** (0.004)	0.016*** (0.004)	0.017*** (0.004)
*Time_Duration*		−0.001 (0.019)	0.002 (0.002)	0.002 (0.002)	0.002 (0.002)
*Ind_Univ_Type*		−0.261 (1.342)	−0.315** (0.104)	−0.255* (0.105)	−0.263* (0.106)
*Att_Univ_985*		−0.661 (1.628)	0.332** (0.121)	0.305* (0.122)	0.271* (0.123)
*Att_Univ_211*		−1.798 (1.292)	−0.160 (0.116)	−0.125 (0.117)	−0.158 (0.118)
*City_GDP*		1.396* (0.598)	0.156*** (0.044)	0.193*** (0.045)	0.222*** (0.046)
*Num_Univ_City*		−0.058* (0.026)	−0.007** (0.002)	−0.007** (0.002)	−0.008*** (0.002)
*N*	110	110	110	110	110
*R*^*2*^	0.537	0.625			
*Adjusted R*^*2*^	0.533	0.578			
*Pseudo R*^*2*^			0.495	0.502	0.507
*AIC*			687.97	680.95	675.59
*BIC*			725.78	721.46	718.79

*p < 0.05; **p < 0.01; ***p < 0.001.

### Moderating effect

To further investigate how new IT utilization (i.e., cloud storage) influences data breaches, we specified an econometric model with cloud storage adoption as a moderating variable. First, we addressed the moderating role of cloud storage in the relationship between vulnerabilities and data breaches. The analytical model was constructed as follows.

6 $$\begin{array}{l}\log \left( {E\left( {Num\_Breach_i{{{\mathrm{|}}}}X_i + Controls} \right)} \right)\\ \quad = \alpha + \beta _1Num\_Vulnerability_i + \beta _2Num\_Research\_Project_i\\ \qquad+\, \beta _3Num\_Data\_Flow_i + \,\beta _4Ind\_Cloud\_Storage_i \\ \qquad+\,\beta _5Num\_Vulnerability_i \ast Ind\_Cloud\_Storage_i + \gamma Controls\end{array}$$

The empirical results are presented in Table 8. Column (1) shows all controls without *Num_Vulnerability, Ind_Cloud_Storage*, and the interaction term. Column (2) is from Column (2) in Table 5 to allow for an easy comparison. Column (3) shows the estimates considering the moderating effect, where the interaction term is negatively related to data breaches (*β* = −0.011, *s.e*. = 0.001, *p* < 0.001). Thus, cloud storage mitigates the positive relationship between vulnerabilities and data breaches as a moderating variable. Cloud storage enables a more integrated consolidation of distributed data stored in different systems, thus making it easier to maintain and manage. Therefore, adopting cloud storage could reduce the possibility of breaches caused by vulnerabilities. Accordingly, H6(a) is supported.

**Table 8:** Moderating effect: vulnerability and cloud storage.

Column	(1)	(2)	(3)
Model	Controls only	No moderating effect	With moderating effect
*Num_Data_Flow*	−0.010 (0.006)	−0.021** (0.008)	−0.016* (0.007)
*Num_Vulnerability*		0.005*** (0.001)	0.015*** (0.001)
*Ind_Cloud_Storage*		−0.335*** (0.088)	0.196 (0.110)
Num_Vulnerability*Ind_Cloud_Storage			−0.011*** (0.001)
*Num_Undergraduate_Major*	0.009*** (0.002)	0.013*** (0.002)	0.011*** (0.002)
*Ind_Training*	−0.500* (0.251)	−0.780** (0.258)	−0.684** (0.255)
*Num_Research_Project*	0.001*** (0.000)	−0.000 (0.000)	0.000 (0.000)
*Num_Master_Program*	0.001** (0.000)	−0.001 (0.001)	−0.001* (0.001)
*Num_Doctoral_Program*	−0.003*** (0.001)	0.000 (0.001)	0.000 (0.001)
*Num_Key_Disciplines*	−0.004 (0.004)	0.017*** (0.004)	0.011* (0.005)
*Time_Duration*	0.004** (0.002)	0.002 (0.002)	0.002 (0.002)
*Ind_Univ_Type*	−0.340** (0.109)	−0.263* (0.106)	−0.124 (0.110)
*Att_Univ_985*	0.523*** (0.116)	0.271* (0.123)	−0.029 (0.130)
*Att_Univ_211*	−0.307** (0.116)	−0.158 (0.118)	−0.150 (0.118)
*City_GDP*	0.228*** (0.044)	0.222*** (0.046)	0.248*** (0.046)
*Num_Univ_City*	−0.011*** (0.002)	−0.008*** (0.002)	−0.006** (0.002)
*Sample Size*	110	110	110
*Pseudo R*^*2*^	0.415	0.507	0.553
*AIC*	789.3	675.6	617.5
*BIC*	827.1	718.8	663.4

*p < 0.05; **p < 0.01; ***p < 0.001.

Below, we addressed the moderating role of cloud storage in the relationship between cross-border data flow and data breaches.

7 $$\begin{array}{l}\log \left( {E\left( {Num\_Breach_i\left| {X_i} \right. + Controls} \right)} \right)\\ \quad = \alpha + \beta _1Num\_Vulnerability_i + \beta _2Num\_Research\_Project_i\\ \qquad+ \,\beta _3Num\_Data\_Flow_i + \beta _4Ind\_Cloud\_Storage_i\\ \qquad+ \,\beta _5Num\_Data\_Flow_i \ast Ind\_Cloud\_Storage_i + \gamma Controls\end{array}$$

Table 9 shows the estimation results. Column (1) represents the controls only, and Columns (2) and (3) report the results without and with a moderating effect, respectively. The interaction term in Column (3) is negatively related to data breaches (*β* = −0.055, *s.e*. = 0.014, *p* < 0.001), indicating that cloud storage strengthens the negative relationship between cross-border data flow and data breaches, as cloud storage makes it easier to transfer data worldwide. Thus, H6(b) is supported.

**Table 9:** Moderating effect: cross-border data flow and cloud storage.

Column	(1)	(2)	(3)
Model	Controls only	No moderating effect	With moderating effect
*Num_Data_Flow*		−0.021** (0.008)	−0.011 (0.008)
*Num_Vulnerability*	0.005*** (0.001)	0.005*** (0.001)	0.008*** (0.001)
*Ind_Cloud_Storage*		−0.335*** (0.088)	−0.182 (0.095)
*Ind_Internet_Trans*		−0.335*** (0.088)	−0.182 (0.095)
Num_Data_Flow*Ind_Cloud_Storage			−0.055*** (0.014)
*Num_Undergraduate_Major*	0.013*** (0.002)	0.013*** (0.002)	0.014*** (0.002)
*Ind_Training*	−0.703** (0.258)	−0.780** (0.258)	−0.740** (0.257)
*Num_Research_Project*	0.000** (0.000)	−0.000 (0.000)	0.000 (0.000)
*Num_Master_Program*	−0.001 (0.000)	−0.001 (0.001)	−0.001 (0.001)
*Num_Doctoral_Program*	−0.000 (0.001)	0.000 (0.001)	−0.000 (0.001)
*Num_Key_Disciplines*	0.012** (0.004)	0.017*** (0.004)	0.015*** (0.004)
*Time_Duration*	0.002 (0.002)	0.002 (0.002)	0.002 (0.002)
*Ind_Univ_Type*	−0.284** (0.104)	−0.263* (0.106)	−0.308** (0.107)
*Att_Univ_985*	0.288* (0.123)	0.271* (0.123)	0.143 (0.126)
*Att_Univ_211*	−0.165 (0.115)	−0.158 (0.118)	−0.147 (0.118)
*City_GDP*	0.173*** (0.045)	0.222*** (0.046)	0.192*** (0.046)
*Num_Univ_City*	−0.007** (0.002)	−0.008*** (0.002)	−0.006** (0.002)
*Sample Size*	110	110	110
*Pseudo R*^*2*^	0.487	0.507	0.521
*AIC*	690.9	675.6	659.8
*BIC*	728.7	718.8	705.8

*p < 0.05; **p < 0.01; ***p < 0.001.

## Robustness checks

To ensure the robustness of the conclusions, this section discusses several robustness checks from four perspectives. First, we tested the significance of the mediating effects. Second, we expanded the time window for several variable measurements to mitigate the impact of COVID-19. Third, we explored whether the effects of specific cloud adoptions differ by redefining the cloud services and classifying them into private and public cloud storage. Finally, because data breach incidents have different risk levels, we considered the effects of various factors at different levels of risk.

### Significance test for mediating effect

Three other methods (Aroian, 1947; Goodman, 1960; Sobel, 1982) were used to test the significance of the mediation effect(s). As shown in Table 10, Row (1) is the test result obtained using Column (1) in Tables 8 and 9 from investigating the relationship between the independent and intermediary variables. Row (2) is for Column (2) in Tables 8 and 9. All *p* values are less than 0.01, except for the Aroian Test of *Num_Data_Flow* in Row (2), where *p* = 0.01006, indicating that the mediation effects are highly significant.

**Table 10:** Robustness check: significance test for mediation effect.

Mediation		Num_Data_Flow				Num_Vulnerability			
Method		Test statistic	Std. error	*p*-value		Test statistic	Std. error	*p*-value	
(1)	Sobel	−2.58571	0.00012	*p* = 0.00972	*p* < 0.01	3.85735	0.00013	*p* = 0.00012	*p* < 0.001
	Aroian	−2.58015	0.00012	*p* = 0.00988	*p* < 0.01	3.82650	0.00013	*p* = 0.00013	*p* < 0.001
	Goodman	−2.59130	0.00012	*p* = 0.00956	*p* < 0.01	3.88897	0.00013	*p* = 0.00010	*p* < 0.001
(2)	Sobel	−2.58004	0.00011	*p* = 0.00988	*p* < 0.01	4.14024	0.00012	*p* = 0.00004	*p* < 0.001
	Aroian	−2.57371	0.00011	*p* = 0.01006	*p* < 0.05	4.11445	0.00012	*p* = 0.00004	*p* < 0.001
	Goodman	−2.58642	0.00011	*p* = 0.00970	*p* < 0.01	4.16652	0.00012	*p* = 0.00003	*p* < 0.001

We then investigated the proportions of the mediation effects and direct effects, as shown in Table 11. Using *Num_Data_Flow* as the mediator, there is a significantly negative mediation effect between the independent and dependent variables. The average direct effects are insignificant. For *Num_Vulnerability* as the mediator, there is a significantly positive mediation effect, and the direct effects are insignificant. This indicates that the effects of vulnerabilities on data breaches going through the mediator account for almost the entire total effects. The mediation and direct effects have different signs, explaining why the proportion of the effects going through the mediator exceeds one.

**Table 11:** Robustness check: bootstrap mediation tests.

MD		Num_Data_Flow			Num_Vulnerability		
Effects		Estimate	95% CI Lower	95% CI Upper	Estimate	95% CI Lower	95% CI Upper
(1)	ACME	−0.00341*	−0.00660	0	0.00348***	0.00231	0
	ADE	−0.00043	−0.00511	0	−0.00035	−0.00309	0
	Total Effect	−0.00384**	−0.00790	0	0.00313**	0.00098	0
	Prop. Mediated	0.92462*	0.18189	3.58	1.08239**	0.60717	3.64
(2)	ACME	−0.00355**	−0.00685	0	0.00358***	0.00236	0.01
	ADE	−0.00055	−0.00474	0	−0.00030	−0.00278	0
	Total Effect	−0.00410*	−0.00870	0	0.00328**	0.00090	0.01
	Prop. Mediated	0.91217*	0.16885	2.85	1.07296**	0.56443	3.57

The number of simulations was 1000, ACME stands for average causal mediation effects, ADE stands for average direct effects, Total Effect stands for the total effect (direct + indirect) of the independent variable on the dependent variable; Prop. Mediated describes the proportion of the effect of the independent variable on the dependent variable that goes through the mediator. *p < 0.05; **p < 0.01; ***p < 0.001.

### Varied length of time window

#### Expansion of the time window length of data breach incidents

For the main analysis, we collected data on breach incidents in 2020. Regarding the global outbreak of COVID-19, the incidents in 2020 may have been affected by fluctuations in the epidemic, making them unrepresentative of typical data security issues in universities. Therefore, the data breach incidents reported during 2017–2019 were used to measure the level of universities’ data security protection.

Column (1) in Table 12 presents the estimated results, where *Num_Breach’* denotes the number of data breach incidents in universities reported during 2017–2019, and *Num_Vulnerability’* measures the number of publicly disclosed vulnerabilities in universities during 2017–2019. Column (3) replicates the original results in Table 5 for ease of comparison. The impacts of the main variables on breaches are consistent, thereby confirming the key findings.

**Table 12:** Robustness check on time window length of data collection.

Column	(1)	(2)	(3)
Dependent Variable	*Num_Breach’*	*Num_Breach*	*Num_Breach*
*Num_Data_Flow*	−0.008 (0.005)		−0.021** (0.008)
*Num_Data_Flow’*		−0.014*** (0.004)	
*Num_Vulnerability*		0.006*** (0.001)	0.005*** (0.001)
*Num_Vulnerability’*	0.004*** (0.000)		
*Ind_Training*	0.511 (0.278)	−0.761** (0.257)	−0.780** (0.258)
*Ind_Cloud_Storage*	−0.217*** (0.054)	−0.287*** (0.085)	−0.335*** (0.088)
*Num_Undergraduate_Major*	0.009*** (0.001)	0.014*** (0.002)	0.013*** (0.002)
*Num_Research_Project*	−0.000 (0.000)	0.000 (0.000)	−0.000 (0.000)
*Num_Master_Program*	−0.001*** (0.000)	−0.001 (0.001)	−0.001 (0.001)
*Num_Doctoral_Program*	0.003*** (0.000)	−0.000 (0.001)	0.000 (0.001)
*Num_Key_Disciplines*	0.017*** (0.002)	0.016*** (0.004)	0.017*** (0.004)
*Time_Duration*	0.002* (0.001)	0.002 (0.002)	0.002 (0.002)
*Ind_Univ_Type*	−0.154* (0.066)	−0.295** (0.106)	−0.263* (0.106)
*Att_Univ_985*	0.037 (0.075)	0.233 (0.124)	0.271* (0.123)
*Att_Univ_211*	−0.280*** (0.073)	−0.137 (0.117)	−0.158 (0.118)
*City_GDP*	0.136*** (0.031)	0.206*** (0.045)	0.222*** (0.046)
*Num_Univ_City*	−0.006*** (0.001)	−0.007*** (0.002)	−0.008*** (0.002)
*Sample Size*	110	110	110
*Pseudo R*^*2*^	0.634	0.510	0.507
*AIC*	903.3	671.6	675.6
*BIC*	946.5	714.8	718.8

Num_Vulnerability: 2020, Num_Vulnerability’: 2017–2019; Num_Breach: 2020, Num_Breach’: 2017–2019; Num_Data_Flow: 2020, Num_Data_Flow’: 2019; *p < 0.05; **p < 0.01; ***p < 0.001.

#### Expansion of the time window length of cross-border data flow

In addition to the COVID-19 outbreak, another breakout in 2020 was related to global medical data sharing, particularly regarding coronavirus epidemic-related data. This may have caused abnormal fluctuations in cross-border data flow at universities. To alleviate this concern, we used the cross-border data flow collected in 2019. Table 12 shows the results in Column (2), where *Num_Data_Flow’* measures the number of cross-border data flows in universities during 2019. The results remain consistent and the significance of *Num_Data_Flow’* is even higher, providing further evidence of the robustness.

### Different cloud service types

As discussed in the empirical results section, adopting cloud storage can result in fewer vulnerabilities and improve data fluidity. However, considering that different types of cloud storage may have different effects, we further classified cloud storage into two types, namely, private and public clouds, as defined by cloud providers in the market. According to Alibaba Cloud, a private cloud provides a corporation or organization with a dedicated cloud environment that can be operated internally by the IT team to better control its computing resources (Li and Li, 2017). A private cloud can be physically located in the organization’s data center or hosted by a service provider. A public cloud is a cloud infrastructure provided by service suppliers for users, individuals, or enterprises. Users can access these servers by purchasing public cloud services and data storage. On a public cloud, all users share the same hardware, storage, and network equipment^8^.

The effects of three variables, *Ind_Cloud_Storage*, *Ind_Cloud_Private*, and *Ind_Cloud_Public*, were investigated. Table 13 shows the results, where *Ind_Cloud_Storage* is coded as “1” if the university adopted any type of cloud storage and “0” otherwise; *Ind_Cloud_Private* is coded as “1” if the university adopted a private cloud and “0” otherwise; and *Ind_Cloud_Public* is coded as “1” if the university adopted a public cloud and “0” otherwise. Notably, public clouds have the most significant negative effects. According to *routine activity theory*, guardianship is essential to cybersecurity, and universities can enhance their guardianship by seeking external governance from external vendors (Pang and Tanriverdi, 2022). Public clouds enable external guardianship provided by cloud service vendors who are more capable of effective information protection (Pang and Tanriverdi, 2022). In addition, outsourcing vendors can achieve economies of scale and scope when offering IT services to clients, making it more economically feasible for vendors with professional security teams to protect their information systems (Levina and Ross, 2003). Therefore, in terms of cloud adoption, public clouds may be a better choice for cybersecurity.

**Table 13:** Robustness check on different cloud types.

Model	(1)	(2)	(3)	(4)	(5)	(6)
*Ind_Cloud_Storage*	−0.335*** (0.088)			−0.530*** (0.126)	−0.215* (0.101)	
*Ind_Cloud_Private*		−0.097 (0.096)		0.315* (0.140)		−0.215* (0.101)
*Ind_Cloud_Public*			−0.460*** (0.121)		−0.315* (0.140)	−0.530*** (0.126)
Other Variables	Included	Included	Included	Included	Included	Included
*Pseudo R*^*2*^	0.507	0.497	0.508	0.511	0.511	0.511
*AIC*	675.6	689.7	675.1	672.4	672.4	672.4
*BIC*	718.8	732.9	718.3	718.3	718.3	718.3

*p < 0.05; ***p < 0.001.

### Different risk levels of data breach

The factors related to the risk of breaches were tested for repercussions beyond the mere occurrence of such breaches. The Education Industry Vulnerability Reporting Platform scores the risks of all data breach incidents on a scale of 0–10. This scale is further categorized as low (0–4), medium (4–7), high (7–9), and severe (9–10) risks. Table 14 presents the descriptive statistics of breaches with different risk levels.

**Table 14:** Descriptive statistics of breaches at different risk levels.

Risk Level	Obs.	Mean	S.D.	Max	Min
Low	110	6.83	8.11	63	0
Medium	110	1.21	2.16	14	0
High	110	0.29	1.14	11	0
Severe	110	0.05	0.25	2	0

Instead of the total number of breaches, the number of breaches with different risk levels was counted for universities in 2020 and regressed onto independent variables. As there were relatively few instances of severe incidents, only three risk levels were considered. The results are presented in Table 15. For low- and medium-risk breach incidents, the cross-border data flow still has a significant negative effect, and the number of publicly disclosed vulnerabilities still has a significant positive effect. The effects of the main variables are highly consistent with the previous research results. For high-risk breach events, a good fit is not achieved because of the small number of observations; however, the coefficient signs of the main variables are consistent. The results show that cross-border data flow only affects the occurrence of medium-risk breaches and that vulnerabilities tend to mostly increase the occurrence of high-risk breaches, as they have the highest significance in the regression results. The adoption of cloud storage may only influence the occurrence of low-risk breaches. Despite the few observations, this finding provides insights into possible future research directions.

**Table 15:** Robustness check on risk levels for data breaches.

Column	(1)	(2)	(3)
Dependent Variable	*Num_Breach_Low*	*Num_Breach_Medium*	*Num_Breach_High*
*Num_Data_Flow*	−0.016 (0.009)	−0.111*** (0.031)	0.069 (0.069)
*Num_Vulnerability*	0.005*** (0.001)	0.009*** (0.002)	0.018*** (0.004)
*Ind_Cloud_Storage*	−0.308** (0.096)	−0.419 (0.238)	−1.464 (0.872)
*Num_Undergraduate_Major*	0.013*** (0.002)	0.007 (0.005)	0.026 (0.017)
*Ind_Training*	−0.718* (0.298)	−1.033 (0.560)	−0.523 (1.686)
*Num_Research_Project*	−0.000 (0.000)	0.001 (0.001)	−0.003* (0.002)
*Num_Master_Program*	−0.000 (0.001)	−0.003 (0.002)	0.001 (0.004)
*Num_Doctoral_Program*	0.000 (0.001)	−0.001 (0.003)	−0.001 (0.007)
*Num_Key_Disciplines*	0.013** (0.005)	0.019 (0.012)	0.055 (0.033)
*Time_Duration*	0.002 (0.002)	0.005 (0.004)	−0.019 (0.011)
*Ind_Univ_Type*	−0.228 (0.116)	−0.469 (0.291)	0.144 (0.739)
*Att_Univ_985*	0.316* (0.134)	0.220 (0.350)	−0.098 (0.805)
*Att_Univ_211*	−0.190 (0.130)	−0.109 (0.326)	−0.318 (0.740)
*City_GDP*	0.204*** (0.051)	0.324** (0.115)	0.210 (0.329)
*Num_Univ_City*	−0.008** (0.002)	−0.016* (0.006)	−0.002 (0.017)
*N*	110	110	110
*Pseudo R*^*2*^	0.459	0.283	0.522
*AIC*	607.1	323.5	120.2
*BIC*	650.3	366.8	163.4

*p < 0.05; **p < 0.01; ***p < 0.001.

## Discussion and conclusion

In this study, we identified and analyzed the key elements of data security incidents in the context of higher education from an empirical perspective. Based on *crime opportunity theory* and *routine activity theory*, we constructed a conceptual model and proposed hypotheses to investigate the underlying mechanisms that impact data breaches. The key findings were obtained through a series of empirical analyses and robustness checks. First, it was determined that the public disclosure of vulnerabilities increased data breaches, which complements the conclusion of Sen and Borle (2015) in the context of universities. Second, when incorporating the cross-border data flow effect and measuring the data fluidity and mobility, we found that it negatively affected data breaches, leading to fewer breaches. Third, academic strength influenced the occurrence of data breaches in different ways. Academically stronger universities tended to have more data flow and publicly reported vulnerabilities, which played a mediating role in the relationship between academic strength and data breaches. Fourth, new information technologies such as cloud storage could help reduce data breaches and have moderating effects on vulnerabilities and data flow. In addition, public clouds were found to be relatively safer than private clouds in terms of data breach issues, which complements the research focusing on cloud services and data securities.

### Theoretical contributions and implications

This study makes theoretical contributions to the literature. First, we contribute to the data security literature by exploring a new context. According to the available literature, this study is among the first to examine the factors influencing data breach risks in the context of universities. Prior studies on data breach risk have focused on several other industries, such as the medical industry and companies. Relatively few studies have focused on universities even though they are at great risk of data breaches. In this study, the increased risk associated with the number of public disclosures of vulnerabilities is highlighted. The underlying mechanisms explaining how academic strength affects the risk of data breaches are investigated.

Second, we contribute to research on data breaches by discussing the effects of cross-border data flow, which are valued and regulated by numerous countries and regions for their contributions to the digital economy and potential security risks. Prior research has barely considered cross-border data flow in the context of data breaches and has mostly focused on developing and managing relevant policies to prevent potential risks incurred by cross-border data flow. We investigate the effects of cross-border data flow on data breaches and provide another perspective for understanding the value of cross-border data flow.

Third, we contribute to the information security literature by identifying the impacts of clouds on data breaches, distinguishing between the effects of different types of cloud adoption on the risk of data breaches. IBM (2022) reported that the cost of a data breach incident in organizations with public, private, or hybrid clouds can differ significantly. Our findings further reveal and strengthen the difference in terms of the impact on data breaches, which has implications for studies focusing on data security in cloud environments. Future research could break down the types of clouds and explore their effects in different contexts to support decision-making relevant to clouds.

Fourth, this study has implications for research on data breaches. Although we focus on a specific industry, and some of the identified factors and key findings are industry specific, they nonetheless provide an impetus for analyzing the causes of data breaches in other contexts, thereby enriching the literature by identifying the factors influencing data security incidents and, in particular, data breaches in the context of universities.

### Practical implications

Our study provides a basis for improving the data security of universities and other scientific research institutions in the higher education industry, which has practical implications for universities aiming to shape their data security strategies to mitigate data security risks. First, regular system maintenance and timely discovery and repair of technical vulnerabilities can reduce opportunities for attackers and create a secure and stable information environment. Second, strengthening data fluidity and openness is conducive to creating more valuable data. Third, when embracing new information technologies, such as cloud storage, universities may consider the possibility of data breaches resulting from different service types, thereby weighing the advantages and disadvantages. Fourth, strengthening the intensity of data security training and improving the data security awareness of relevant personnel can help prevent problems and information breaches caused by human errors before they occur.

### Limitations and future research

Certain limitations and future research directions are summarized as follows. First, the data were confined to universities in China and had a relatively short time series. A dynamic panel integrating data analysis along the time dimension for institutions of higher education in different countries could be empirically created and analyzed in future research. Second, the scale of a data breach was not incorporated in this analysis for an assessment of the risk impact, as the measurements for the numbers and types of leaked data were not accessible from the Education Industry Vulnerability Reporting Platform. Providing risk quantification of data breach incidents could be an important future research direction. Third, higher education institutions invest heavily in IT (Nash, 2007), which plays a key role in data-security management. Thus, the effects of information security investments and new IT utilization, such as biometric identification technologies, could be quantitatively valued in future research.

## Supplementary information

Dataset 1

## Data Availability

The datasets of data breach incidents and disclosed vulnerabilities analyzed during the current study are from the Education Industry Vulnerability Reporting Platform, available at https://src.sjtu.edu.cn/. The data analyzed during this study are included in the supplementary information files. The remainder of the datasets are available from the corresponding author upon reasonable request.

## Footnotes

1 Statista provides annual number of data compromises and individuals impacted in the United States from 2005 to 2022. See https://www.statista.com/statistics/273550/data-breaches-recorded-in-the-united-states-by-number-of-breaches-and-records-exposed (Accessed 10 Feb 2023)

2 Private Rights Clearinghouse provides a chronology of data breaches. See https://privacyrights.org/data-breaches (Accessed 29 March 2023)

3 The details of the Arden University data breach are provided by the Group Action Lawyers. See https://www.groupactionlawyers.co.uk/blog/arden-university-data-breach-group-compensation-action (Accessed 7 Feb 2023)

4 Information about the Education Industry Vulnerability Reporting Platform can be found on https://src.sjtu.edu.cn/ (Accessed 7 Feb 2023)

5 The details of the context-aware security defined by Gartner can be found on http://www.Gartner.com/IT-glossary/context-aware-security (Accessed 7 Feb 2023)

6 The “Project 985” is a construction project to build a number of world-class universities and a number of internationally renowned high-level research universities. Normally, they are regarded as the top universities in China. The “Project 211” focuses on the construction of approximately 100 higher education institutions and several key disciplines. Note that the 985 project universities are included in the list of the 211 project universities.

7 First-tier cities include Beijing, Shanghai, Guangzhou and Shenzhen. New first-tier cities include Chengdu, Chongqing, Hangzhou, Wuhan, Xi’an, Tianjin, Suzhou, Nanjing, Zhengzhou, Changsha, Dongguan, Shenyang, Qingdao, Hefei and Foshan, according to the list of new first-tier cities selected by YiMagazine in 2020.

8 Alibaba Cloud provides the definitions of private and public clouds. See https://www.alibabacloud.com/zh/knowledge/what-is-private-cloud and https://www.alibabacloud.com/zh/knowledge/what-is-public-cloud (Accessed 7 Feb 2023)
